# Influence of sleep disorders on the behavior of individuals with autism spectrum disorder

**DOI:** 10.3389/fnhum.2015.00347

**Published:** 2015-06-18

**Authors:** Cintia C. Fadini, Dionísia A. Lamônica, Agnes C. Fett-Conte, Elaine Osório, Gabriela M. Zuculo, Célia M. Giacheti, Luciana Pinato

**Affiliations:** ^1^Department of Speech, Language and Hearing Therapy, São Paulo State University-UNESPMarília, SP, Brazil; ^2^Department of Speech, Language and Hearing Therapy, University of São PauloBauru, SP, Brazil; ^3^Faculdade de Medicina de São José do Rio Preto (FAMERP), São José do Rio PretoSP, Brazil

**Keywords:** sleep, behavior, autism spectrum disorder, communication, social interaction

## Abstract

The aim of this study was to investigate the correlation between sleep disorders and the behavior of subjects with autism spectrum disorder (ASD) and control subjects using specific questionnaires. A small percentage (1.8%) of the control subjects had symptoms indicative of sleep-breathing disorders (SBD) and nocturnal sweating. Fifty-nine percent of the subjects with ASD had symptoms indicative of at least one sleep disorder, with SBD the most commonly reported (38%). In the control group, the symptoms of SBD were correlated with social, thought, attentional, aggression, externalizing and behavioral problems. In the ASD group, disorders of arousal (DA) were correlated with thinking problems, and disorders of excessive somnolence were correlated with thinking and behavioral problems. These results suggest that children and adolescents with ASD have a high frequency of sleep disorders, which in turn correlate with some of the behavioral traits that they already exhibit. Furthermore, sleep disturbances, when present in the typically developing children, also correlated with behavioral problems.

## Introduction

Autism spectrum disorder (ASD) is a persistent condition that affects communication and social interaction across multiple contexts, and it includes the presence of atypical and heterogeneous behaviors that manifest with different degrees of severity. The degree of severity is assigned with respect to the difficulties and restrictions in the skills of social communication and repetitive behavior models. Some individuals use speech to communicate, but others are not able to communicate through spoken language (American Psychiatric Association – APA, 2013).

The symptomatology of ASD highlights the high prevalence (40–86%) of severe disorders of the sleep-wake cycle (Richdale, 1999; Fombonne, 2005; Liu et al., 2006; Richdale and Schreck, 2009; Maski and Kothare, 2013; Humphreys et al., 2014), which in recent years, has led to growing research on the causes and consequences of these disorders as well as possible treatments for them. Regarding the possible causes, there is a hypothesis that children who are more sensitive to external stimuli can become more vigilant and hyperactive and have some resistance at bedtime, increasing insomnia rates in this population (Hollway and Aman, 2011). Furthermore, this rhythm is known to be modulated by the neurohormone melatonin, the nocturnal production of which is decreased in autism and which is good a candidate for sleep treatment (Cortesi et al., 2012; Tordjman et al., 2015)

Regarding the sleep characteristics, subjective data about sleep patterns, such as questionnaires completed by the parents and sleep diaries, as well as objective data (polysomnography and actigraphy) indicate that the most striking feature of sleep in individuals with ASD is the inability to maintain the required latency time; common complaints include difficulty initiating and/or maintaining sleep, reduced total sleep, waking during the night and in the early morning and being unable to stay awake during the day (Elia et al., 2000; Schreck and Mulick, 2000; Miano and Ferri, 2010).

As a consequence, the sleep–wake rhythm associated with biological circadian rhythms can be viewed as an adaptation to the day–night cycle, which seems to be important for the rhythmicity and synchrony of motor, emotional, and relational rhythms during the early development of social communication (Tordjman et al., 2015). Sleep problems are thus known to be correlated with metabolic, immune, and behavior disorders; attention deficits (Sadeh et al., 2006; Golombek et al., 2013); anxiety and depression (Forbes et al., 2008); and hyperactivity and impulsivity (Touchette et al., 2009). Thus, sleep disturbances, whether transient or permanent, could become more severe clinical conditions in individuals with ASD and interfere with the behavioral aspects of attention and learning (Johnson et al., 2009). Furthermore, there is a direct relationship between a reduction in the hours of night sleep and the severity of behavioral stereotypes, difficulties in social interactions, communication disorders and family stress (Richdale, 1999; Elia et al., 2000; Gregory and O’Connor, 2002; Thunström, 2002; O’Brien and Gozal, 2004; Hoffman et al., 2008; Miano and Ferri, 2010; Minkel et al., 2012).

Because several studies have indicated that sleep quality influences aspects of behavior (Malow et al., 2006; Giannotti et al., 2008; Maski and Kothare, 2013) and that the treatment of sleep disturbances can improve daytime behavior, including less irritability, anxiety, withdrawal and hyperactivity; fewer affective problems, attention deficits, and stereotyped and compulsive behaviors; and better mood and communication, in children with ASD and in children who are neurologically multiply disabled (Jan et al., 1994; Horrigan and Barnhill, 1997; Ishizaki et al., 1999; Paavonen et al., 2003; Garstang and Wallis, 2006; Giannotti et al., 2006; De Leersnyder et al., 2011; Wright et al., 2011; Malow et al., 2012), the aim of this study was to investigate the patterns of sleep, behavioral profiles and the correlation between sleep disorders and behavior in children and adolescents with ASD.

This study provided pilot data toward a new study, in which we intend to expand the sample, use pharmacological treatment to improve sleep quality in this population and investigate whether there was consequent improvement in behavioral and cognitive parameters.

## Materials and Methods

This was a cross-sectional clinical study in accordance with the Regulatory Norms on Human Research. The study protocol was approved by the ethics committee of the local institution (proc. 1001/2011), and informed consent was obtained from the parents of the participants prior to the start of data collection.

### Characterization of the Subjects

The study included 101 subjects, who were divided into two groups: the ASD group (*n* = 45) and the control group (*n* = 56). The criteria for inclusion in the ASD group included a diagnosis of ASD (via a medical report; American Psychiatric Association – APA, 2013) based on the DSM-V criteria; age between 4 and 18 years (either gender); the absence of clinical signs suggestive of known monogenic genetic diseases; the absence of chromosomal abnormalities or multifactorial diseases; and the absence of mutations of the FMR1 gene.

To complement the strong clinical consensus that the child truly had an ASD diagnosis and homogenize the sample of subjects, the Scale for the Assessment of Autistic Behavior (ATA) and the Childhood Autism Rating Scale (CARS; Assumpção et al., 1999; Pereira, 2007) were used, in addition to clinical and laboratory genetic assessments and an evaluation of spoken language. The ATA uses the score of 15 as a cutoff. Subjects who show values equal to or greater than 15 are considered to have ASD.

The CARS evaluates behavior in 14 domains instead of one general category of autism impression. The scores range from 15–60, and the cutoff for autism is 30. Scores between 30 and 36 indicate mild to moderate symptoms, whereas scores above 37 indicate severe symptoms.

Genetic tests were performed in all of the participants in the ASD group and consisted of clinical and karyotype analyses using the GTG staining technique (resolution between 450 and 550 bands), with 20 metaphases analyzed. FMR1 gene mutations were also assessed by analyzing DNA from peripheral blood.

The evaluation of spoken language was performed by observing communicative behavior (expression and reception of spoken language), and subjects were classified as verbal or non-verbal. Individuals who communicated either by echolalia, single words or simple phrases (52%) were classified as “verbal”, and individuals with mutism, or who produced sounds/jargon without communicative intent (48%) were classified as “non-verbal” with regard to spoken language.

During the recruitment process, 75 children with clinical diagnoses of ASD were initially recruited through parental contact in specialized institutions. Of these, 30 were excluded because did not comply with the inclusion criteria described above.

The criteria for inclusion in the control group consisted of typical development and age between 4 and 18 years. The control subjects were recruited through local schools as consecutive cases, trying to achieve pairing with the ASD group.

The ASD group consisted of 45 individuals of both genders who were diagnosed with ASD and who were without comorbidities. Of these, 78% were male and 22% were female. The age of these participants ranged from 4 to 18 years (mean 9.7 ± 4.1). The mean ATA scale value was 33 ± 8.1. With respect to socioeconomic levels, 50% of the subjects belonged to the social class B2, 20% belonged to B1, 20% belonged to C1, and 10% belonged to A2.

The control group consisted of 56 subjects of both genders: 77% male and 23% female. The age of these participants ranged from 4 to 18 years (mean 10.1 ± 3.9). Individuals in this group scored fewer than 15 points on the ATA scale and scored below 30 points on the CARS scale. With respect to socioeconomic levels, 45% of the subjects belonged to the social class B2, 20% belonged to B1, 5% belonged to C2, 20% belonged to C1, and 10% belonged to A2. All of the study procedures were conducted without the participants of the ASD group discontinuing the use of medications for behavioral seizures and anxiety (77% of the ASD group used some type of medication). In the control group, none of the participants used medication for these purposes. The participants in the ASD group received regular or specialized education in addition to therapeutic procedures in specialized institutions with a multidisciplinary approach. Individuals in the control group received regular education at public or private schools.

### Sleep Disturbance Scale for Children (SDSC)

The Sleep Disturbance Scale for Children (SDSC; Assumpção et al., 1999) is a questionnaire that contains 26 items for the assessment of sleep in children and adolescents aged 3–18 years. Each item is scored from 1 (never) to 5 (always), according to its frequency in the last 6 weeks; thus, higher numerical values reflect a greater severity of clinical symptoms. The SDSC contains six subscales: ***disorders of initiating and maintaining sleep—DIMS*** (including sleep duration, sleep latency, going to bed without being sleepy, difficulty sleeping, sleep without anxiety, nocturnal awakenings and difficulty sleeping); ***sleep-breathing disorders—SBD*** (including breathing difficulties, sleep apnea and snoring); ***disorders of arousal—DA*** (including sleepwalking, sleep terrors, and nightmares); ***sleep–wake transition disorders—SWTD*** (including hypnic jerks, rhythmic movement disorders, hypnagogic hallucinations, nocturnal hyperkinesias, and bruxism); ***disorders of excessive somnolence—DES*** (including difficulty waking up, waking up tired, sleep paralysis and daytime sleepiness); and ***sleep hyperhydrosis—SHY*** (including sweating during sleep and perspiring during the night). Furthermore, the sum of the scores provides an overall score of sleep, with a possible range from 26–130 points; the average scores were compared between the two groups. This questionnaire was applied through a direct interview with the parents.

### Analysis of Child Behavior

The behavioral profiles of the individuals with ASD and their respective controls were obtained from the *“Child Behavior Checklist for ages 4-18”* (CBCL/4-18; Achenbach, 1991), Portuguese version, as normed by Bordin et al. (1995). In behavioral analyses, the CBCL is an instrument that is often used to track behavioral problems in individuals with ASD (Ivanova et al., 2007; Kuusikko et al., 2008; Marteletto et al., 2011; Mazefsky et al., 2011; Park et al., 2014).

This questionnaire was applied via a direct interview with the father or mother and consists of 113 items related to behavioral problems, in which the informant classifies the behavior as not true or absent (score = 0); partially or sometimes true (score = 1); or very true or often true (score = 2) over the last 6 months. The sum of the scores allows the evaluator to draw a behavioral profile of the child or adolescent (internalizing problems or externalizing problems) derived from an analysis of eight groupings of items: anxiety/depression, attention problems, delinquent behavior, social problems, thought problems, withdrawal, somatic complaints and aggressive behavior.

The raw scores of each factor were transformed into T scores based on the published norms. These scores suggested three levels, representing unaffected individuals to the most severely affected individuals, with symptoms ranging from non-clinical to clinical, respectively. The score for the non-clinical category is less than 67; the score for the borderline category is 67–70, inclusive; and the score for the clinical category is greater than 70. For internalizing and externalizing problems, this ratio should be less than 60 for the non-clinical category, from 60–63 for the borderline category and greater than 63 for the clinical category.

Both questionnaires were completed by the main caregivers of the children. To identify the participants’ socioeconomic levels, a questionnaire proposed by the Brazilian Association of Research Companies Associação Brasileira de Empresas de Pesquisa – ABEP (2003) was used. Following their criteria, the socioeconomic classes ranged from A to E, representing higher to lower income levels, respectively Associação Brasileira de Empresas de Pesquisa – ABEP (2003).

### Statistical Analysis

The data regarding the presence of sleep disturbances and clinical behaviors were expressed as percentages. The correlations between all the SDSC and CBCL scores and their subscales were analyzed using a Spearman correlation test. The results are expressed with a 95% CI. Statistical analysis was performed using Prism 5.0 software for Windows (GraphPad Software, Inc., CA, USA).

## Results

**Figure 1:**
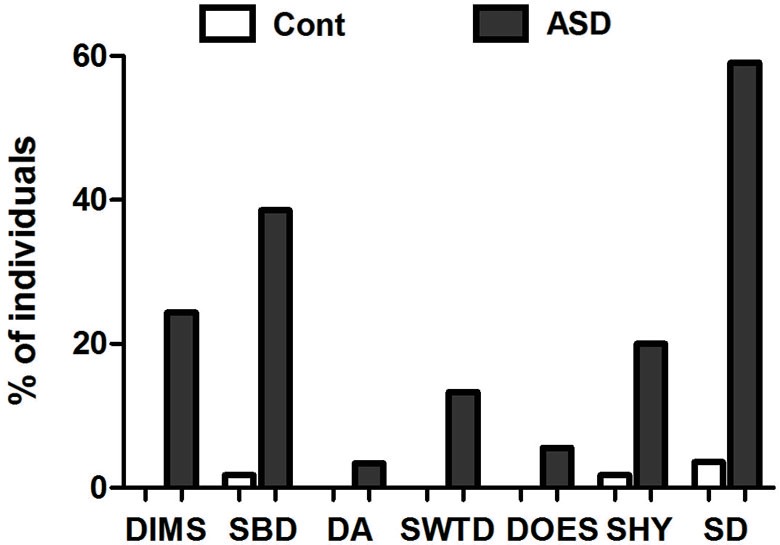
**Percentage of individuals experiencing sleep disturbances**. Distribution in percentage (%) of subjects in the control group and the group with autism spectrum disorder (ASD) who had sleep disorders according to the Sleep Disturbance Scale for Children (SDSC), within the following sub-items: disorders of initiating and maintaining sleep—DIMS, sleep-breathing disorders—SBD, disorders of arousal—DA, sleep-wake transition disorders—SWTD, disorders of excessive somnolence—DES and sleep hyperhydrosis—SHY. *N* = 56 in the control group and 45 in the ASD group.

The results of the SDSC showed that 59% of the individuals with ASD had indicators of at least one type of sleep disorder. Among the six subscales of sleep disorders proposed, indicators of SBD were the most prevalent in the ASD population (38%). In addition, DIMS were present in 24% of the subjects, SWTD were present in 13% of the subjects, and SHY was present in 20% of the subjects in the ASD group. DA (3.4%) and DES (5.6%) appeared at a lower rate in the ASD group. Conversely, in the control group, 1.8% of individuals had indicators for SBD, and 1.8% had indicators for SHY (Figure 1; Table 1).

**Table 1:** **Percentage of individuals experiencing sleep disturbances according to Sleep Disturbance Scale for Children (SDSC)**.

	Sleep disorders according to SDSC													
	DIM		SBD		DA		SWTD		DES		SHY		TS	
	Control	ASD	Control	ASD	Control	ASD	Control	ASD	Control	ASD	Control	ASD	Control	ASD
Mean	11.9	15.9*	4.5	5.6	3.5	3.2	10.1	8.9	7.6	8.4	3.4	4.8*	37.8	47.8*
Standard error	0.5	0.9	0.2	0.4	0.2	0.1	0.7	0.6	0.4	0.6	0.2	0.4	1.2	1.6
Cut off	>21	>21	>6	>6	>7	>7	>15	>15	>19	>19	>7	>7	>52	>52
Pathological (%)	0	23.7	1.8	37.7	0	3.4	0	13	0	5.6	1.8	20	3.6	40

Mean ± standard error and cut off of the SDSC scores and distribution in percentage (%) of subjects in the control group and the group with autism spectrum disorder (ASD) who had DIMS—disorders of initiating and maintaining sleep; SBD—sleep-breathing disorders; DA—disorders of arousal; SWTD—sleep–wake transition disorders; DES—disorders of excessive somnolence; SHY—sleep hyperhidrosis; TS—overall score. *p < 0.05.

**Figure 2:**
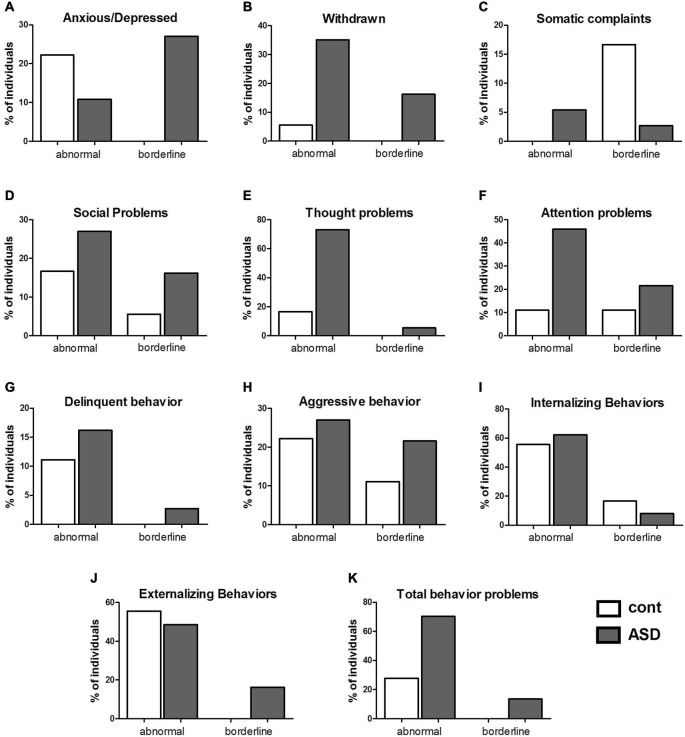
**Comparison between controls and individuals with ASD with clinical (abnormal) or borderline values in the scores for behavioral problems**. Percentage (%) of subjects with clinical or borderline scores in each scale of behavior found in the Child Behavior Check List (CBCL) in the control and ASD groups. In **(A)**, anxious/depressed; In **(B)**, withdrawn; In **(C)**, somatic complaints; In **(D)**, social problems; In **(E)**, thought problems; In **(F)**, attention problems; In **(G)**, delinquent behavior; In **(H)**, aggressive behavior; In **(I)**, internalizing problems. *N* = 56 in the control group and 45 in the ASD group; In **(J)**, externalizing problems; and in **(K)**, total behavior problems.

In the control group, the percentage of subjects in the clinical category included 22% for anxious/depressed problems (Figure 2A), 5% were withdrawn (Figure 2B), 0% had somatic complaints (Figure 2C), 17% had social problems (Figure 2D), 17% had thought problems (Figure 2E), 11% had attention problems (Figure 2F), 11% showed delinquent behavior (Figure 2G), and 22% showed aggressive behavior (Figure 2H). When the categories were grouped, 55% of the subjects in the control group were classified in the clinical category for internalizing problems (Figure 2I), 55% had externalizing problems (Figure 2J), and 27% were classified in the clinical category for total behavioral problems (Figure 2K).

For the other categories of the CBCL classification, the percentage of subjects who achieved a borderline category included: for anxious/depressed problems, 27% of the ASD group and 0% of the control group (Figure 2A); for withdrawal problems, 16% of the ASD group and 0% of the control group (Figure 2B); for somatic complaints, 3% of the ASD group and 17% of the control group (Figure 2C); for social problems, 16% of the ASD group and 5% of the control group (Figure 2D); for thought problems, 5% of the ASD group and 0% of the control group (Figure 2E); for attention problems, 22% of the ASD group and 11% of the control group (Figure 2F); for delinquent behavior, 3% of the ASD group and 0% of the control group (Figure 2G); and for aggressive behavior, 22% of the ASD group and 11% of the control group (Figure 2H). When the categories were grouped, 8% of the ASD group and 16% of the control group were classified as borderline for internalizing problems (Figure 2I), 16% of the ASD group and 0% of the control group were classified as borderline for externalizing problems (Figure 2J), and 13% of the ASD and 0% of the control group were classified as borderline for total behavioral problems (Figure 2K).

**Figure 3:**
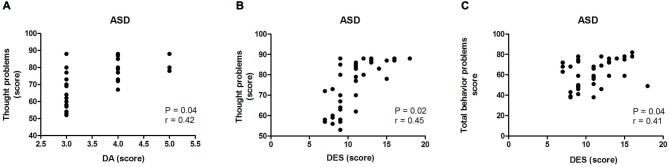
**Correlation between the scores on the Disturbance Scale for Children (SDSC) and the Child Behavior Check List (CBCL) in the ASD group**. In **(A)** correlation between disorders of arousal—DA—and thought problems in the ASD group (*p* < 0.05; *r* = 0.42); In **(B)** correlation between disorders of excessive somnolence—DES—and thought problems in the ASD group (*p* < 0.05; *r* = 0.45); In **(C)** correlation between disorders of excessive somnolence—DES—and behavioral problems in the ASD group (*p* < 0.05; *r* = 0.41).

In the analysis of correlations between all the results of the SDSC and the CBCL, we found a correlation between DA and thought problems (*p* = 0.04, *r* = 0.42; Figure 3A), between DES and thought problems (*p* = 0.02, *r* = 0.45; Figure 3B) and between DES and total behavioral problems (*p* = 0.04, *r* = 0.41; Figure 3C) in the ASD group. Thus, the correlation analysis showed that higher DA scores corresponded to more severe disturbances of thought. In addition, higher DES scores corresponded to more severe thought and total behavioral problems in the ASD group. No correlation between scores of all others parameters of sleep and behavior was found in the multiple comparisons.

## Discussion

The results showed that 59% of the ASD group had indicators of at least of one type of sleep disorder, which agrees with the prevalence reported in the literature, which estimates that 44–83% of individuals experience sleep disorders in this population (Richdale, 1999; Wiggs and Stores, 2004; Johnson et al., 2009; Richdale and Schreck, 2009). This value was higher than that of the control group, in which only 3.6% of the participants exhibited indicators of sleep disturbances. The prevalence of sleep disorders in the control population varies widely in the literature: between 5 and 40% of typically developing individuals experience sleep disorders (Bruni et al., 1996; Ivanenko and Gururaj, 2009).

Among the six subscales of sleep disorders, all were present in different percentages in the ASD group. The indicators of SBD, DIMS, SWTD and SH were the most prevalent in the ASD group. These data are in agreement with the values reported in the literature. In addition, frequent complaints of snoring and nocturnal sweating and an increased frequency and longer periods of nocturnal awakenings have been reported (Richdale, 1999; Richdale and Schreck, 2009; Taylor et al., 2012).

Compared with the control group, higher values, on average, were found for difficulties in initiating or maintaining sleep, SH and total sleep problems in the ASD group. For the other disorders, the group averages of the values showed no significant differences, possibly due to the heterogeneous profile of the group of individuals with ASD who showed varying sleep patterns. Another fact that needs to be considered is the use of medication by the ASD group (77%). Drug treatments for behavioral seizures and anxiety, among other disorders, may cause side effects such as sedation, drowsiness or insomnia (Rotta, 2002; Dorris et al., 2008), which can obscure the diagnosis of sleep disorders (Miano and Ferri, 2010). There is a trend for typically developing children to overcome sleep problems over the course of aging. In the present study, this issue was not evaluated, but it has been demonstrated that individuals with ASD seem less likely to overcome sleep problems over time (Hodge et al., 2014).

The behavioral profiles of the individuals with ASD were characterized by behavioral problems such as social anxiety and depression; withdrawal; and somatic, thought, attention, delinquency, aggression, internalizing, externalizing, and total behavioral problems, which corroborates previous data (Kobayashi and Murata, 1998; Bölte et al., 1999; Marteletto et al., 2011) and the characteristics that the DSM V presents for ASD. In the control group, with the exception of anxiety disorders and depression and externalizing problems, all of the other behavioral problems were either found at a lower rate compared with the ASD group or were absent.

Regarding the possible relationships between behavior and sleep disturbances, as demonstrated in children without developmental problems (Paavonen et al., 2009), correlation tests showed that sleep disorders were correlated with behavioral problems in the ASD group in the present study. It was expected that the presence of these disorders could negatively influence intrinsic ASD characteristics, especially with regard to cognition and behavior (Elia et al., 2000; Taylor et al., 2012; Schwichtenberg et al., 2013). The results showed that an arousal disorder and excessive daytime sleepiness were the two disorders that were correlated with behavioral problems in the ASD group.

The finding of a correlation between sleep disturbances and behavior in individuals with ASD in this study is preliminary and does not determine causality, but there is evidence that improving sleep quality positively influences behavioral contexts (Vriend et al., 2011). In the interpretation of these results is necessary to consider that these correlations were performed in a single test and are not corrected for multiple comparisons. It is necessary to consider the limitations of these analyses. Further studies with a larger number of individuals with ASD and that evaluate behavior before and after treatment to improve sleep patterns are still needed to clarify this correlation and will be the next step of this study.

## Conclusions

The population with ASD showed a high rate of sleep disorders, the most frequent indicators included those of respiratory disorders, disorders related to the onset and maintenance of sleep, hyperhydrosis and disorders affecting the transition from wakefulness to sleep. In addition, the individuals with ASD showed arousal disorders and excessive daytime sleepiness more often than the control subjects.

The individuals with ASD showed all of the behavioral problems suggested in the CBCL test, and more frequently showed thought and attention problems as well as withdrawal. Compared with the control group, the ASD group showed a higher percentage of behavioral problems, except for problems of anxiety and depression and externalizing problems.

Higher sleep disorder scores correlated with behavioral problems in both groups. Specifically in the ASD group, higher scores for DA and excessive daytime sleepiness were related with worse thought and total behavioral problems.

## Conflict of Interest Statement

The authors declare that the research was conducted in the absence of any commercial or financial relationships that could be construed as a potential conflict of interest.
